# A Framework for Mobilizing Health Care to Respond to the Community Within the COVID-19 Pandemic

**DOI:** 10.5888/pcd18.200572

**Published:** 2021-04-01

**Authors:** Fayron Epps, Zanthia Wiley, Larissa J. Teunis, Theodore M. Johnson, Rachel E. Patzer, Igho Ofotokun, Nicole Franks

**Affiliations:** 1 Nell Hodgson Woodruff School of Nursing, Emory University, Atlanta, Georgia; 2 School of Medicine, Emory University, Atlanta, Georgia

## Abstract

Cultural mistrust of government with regard to health issues has pressed the need to engage trusted community leaders with influence and reach in disproportionately affected communities to ensure that essential public health activities related to COVID-19 occur among populations experiencing disproportionate impact from the pandemic. In April of 2020, a Georgia-based integrated academic health care system created a Community Outreach and Health Disparities Collaborative to unite trusted community leaders from faith-based, civic, and health-sector organizations to work with the health system and Emory University to develop tailored approaches and mobilize support within the context of the communities’ cultural and individual needs to reduce the burden of COVID-19. We describe the framework used to join health care and academic collaborators with community partners to mobilize efforts to address the disproportionate impact of COVID-19 on racial, ethnic, and socioeconomic minority groups. The framework outlines a series of steps taken that led to a community-driven collaboration designed to engage local influential community leaders as partners in improving access to care for disproportionately affected communities, collaborations that could be replicated by other large health care systems. This framework can also be applied to other chronic diseases or future public health emergencies to improve communication, education, and health care access for communities experiencing disproportionate impact.

**Summary**

**What is already known on this topic?**

The COVID-19 pandemic is a pressing public health challenge. Interventions to reduce disparities in disease such as COVID-19 between racial and ethnic populations are most effective when they are multifactorial and community-based.

**What is added by this report?**

We hypothesize that a framework of delivering health information through trusted community leaders may partially mitigate the deleterious effects of government mistrust and could increase COVID-19 testing.

**What are the implications for public health practice?**

Although specifically directed toward the COVID-19 crisis, this particular framework could be useful in addressing the disproportionate share of chronic and other diseases among racial or ethnic minority populations and other disproportionately affected populations.

## Problem

The COVID-19 pandemic has amplified health care disparities, differences in social determinants of health, and cultural mistrust that have historically and negatively affected racial and ethnic minority communities and their access to equitable health care (1). With the spread of COVID-19 in our communities, we are acutely aware that Black, Hispanic/Latino, and rural communities are disproportionately affected by the virus. Our proposed work is based in Georgia, one of the early COVID‐19 epicenters in the United States that has high rates of racial and ethnic inequality in income, housing, and disease burden (2–4), yet was also the first state to “re‐open” despite public health advice to the contrary. One study found that although only 32% of residents in metropolitian Atlanta are Black, 79% of COVID-19 patients hospitalized at 6 metropolitian Atlanta hospitals from March to April 2020 were Black, and diabetes, hypertension, chronic kidney disease, obesity, and smoking were all more prevalent among hospitalized patients than among nonhospitalized patients (5). The COVID-19 Health Equity Interactive Dashboard, sponsored by our academic collaborators at Emory University, also highlighted the substantial health care disparities among COVID-19 patients in Georgia with corresponding effects on racial and ethnic minority populations (6).

Structural racism refers to manners in which societies foster racial discrimination in areas such as housing, employment, and health care. Examples of how social determinants of health affect racial minority communities during this pandemic include the inability for many to effectively socially distance in multigenerational households, the inability for frontline employees to work from home, the lack of health care insurance, and the lack of access to COVID-19 testing sites (7). Evidence of a positive association of stay-at-home orders with lower state-level COVID-19 case rates exists, and states with larger Black populations have higher rates of COVID-19 than states with smaller Black populations, which highlights the importance of these stay-at-home orders in addressing racial disparities in COVID-19 rates of infection (8).

There is a deep-rooted history of cultural mistrust in the United States that stems from numerous atrocities to racial minority communities, such as the well-known US Public Health Service syphilis study performed at Tuskegee and not so well-known incidents, including gynecologic procedures on enslaved Black women and grave-robbing in slave quarters with resultant unauthorized autopsies (9,10). Fear of deportation by the US Immigration and Customs Enforcement is also a consideration in Hispanic/Latino communities (1). This mistrust not only serves as a barrier for accessing health care but also in the consideration of enrollment in clinical trials.

Disparities in health care access and patient outcomes are associated with factors such as race, gender identity, sexual orientation, primary language, and socioeconomic status. The structural and cultural divide between the academic health care systems and underrepresented minority communities must be bridged by trusted sources that, in many cases, are community organizations. Community organizations that are faith-based, nonprofit, civic, social support–related, or education-focused serve as the foundation of community engagement. Because many Black people and people in other underrepresented minority groups are often not included in health decision making and policy development, we developed an integrated academic health care system and community coalition to address complex health challenges and stretch and test the capacity of traditional health systems to reduce the burden of COVID-19. This article describes the development of this coalition.

## Call to Action

### Coalition recruitment

The chair of the Emory Healthcare board of trustees charged 2 health care board members to lead a group of clinicians, researchers, educators, and health advocates to mitigate disparities and determine how the health care system could play a role in reducing the disproportionate burden of COVID-19. This charge led to the development of the Community Outreach and Health Disparities Collaborative (Table 1). This team comprised health care professionals and leaders from the disciplines of pharmacy, public health, nursing, medicine, and health care operations. A major initiative of the team was to unite trusted community leaders from faith-based, nonprofit, education, civic, and health sector organizations to work with the health care system and university to develop tailored approaches and mobilize support within the context of the communities’ cultural and individual needs to reduce the burden of COVID-19. A steering committee was established and created 4 work groups to achieve the goals of the team: 1) messaging — aimed at developing targeted message content and engaging community representatives, such as social influencers and academic experts, to provide education and promote best practices for COVID-19 prevention and intervention; 2) research — focused on advancing new knowledge and scholarship in health disparities specific to COVID-19; 3) data — integrates data sources to analyze and identify populations experiencing disproportionate impact in order to direct resources and target interventions; and 4) community partnerships — connecting with trusted community leaders (faith-based, civic and other organizations, businesses, and others) for bidirectional learning, feedback, and collaborative interventions.

**Table 1:** Community Outreach and Health Disparities Collaborative Framework for Mobilizing Health Care to Respond to the Community During the COVID-19 Pandemic

Element	Governance	Messaging and Education	Data	Research	Community Partnerships
Goal	Develop and implement a strategy for the collaborative to address the disproportionate impact of COVID-19 on the health system patient service areas	Tailor public-facing education engagements and materials for disproportionately impacted communities Establish a speaker’s bureau of messengers that reflect the community served	Identify COVID-19–impacted community hot spots and blind spots Develop reporting strategy to guide current and future disparity awareness	Develop and implement a research platform to publish findings and interventions of the collaborative	Establish a community partner advisory board for bidirectional learning and feedback
Input	Members: health system president, board of trustees, senior academic leader, senior health system leader, innovation leader, health system data analytics leader, community leader(s)	Subject matter experts on health equity, health disparities, infectious diseases, chronic disease, public health, vaccination, community outreach, COVID-19 Social influencers and marketing expertise for targeted communities	Public health data, health system data, and research data collaborators Data streams: symptom, race, ethnicity, age, language, comorbidity, social determinants of health	Health disparities researchers	Community partners and leaders
Output	Steering committee with 4 work groups Strategic plan Health system resource support	Virtual and in-person education events Messaging campaigns on all platforms — print/radio/social media Speaker’s bureau	Dashboard illustrating chronic disease, health disparity, and social determinants data Continuous analysis and recommendation of hot spot and blind spot interventions	Assess and promote health disparities research Develop COVID-19 health disparities research questions and shared learning framework	Advisory meetings Education events
Metrics	Steering committee engagement Work group goals met Amount of secured funding	Number of messengers reflecting the community Impact of messaging campaigns on platforms — print/radio/social media Attendance of education events	Improvement of identified health disparity gap	Number of grant-supported health disparities and COVID-19 projects Number of published health disparities and COVID-19 projects	Survey participation Meeting attendance

The president of the health system was tapped as the executive sponsor of the collaborative to ensure connection to the health system’s COVID-19 response efforts and accountability to the directive of the board of trustees. Weekly reports were made to the collaborative’s COVID-19 Incident Command Center, and quarterly reports were made to the board. The established steering committee comprised the board of trustees’ cochairs, senior university and health system leaders, innovation and data experts, public health department representatives, and a leader of a local medical association for Black physicians. They met weekly to develop the strategic work plan, engage participants, and evaluate sources of funding to support intervention efforts. After 3 months, the steering committee added a health system director of community engagement and began to field requests for COVID-19 program support partnerships. The steering committee provided the key benefit of centralized leadership, decision making, triage of requests for community partnerships, and a vector of escalation of barriers to the Incident Command Center for mitigation.

The messaging work group comprised university and health care educators with marketing and communication system resources. This group developed segmented marketing strategies to leverage messengers representative of the community as a trusted voice to educate and inform communities of focus and community leaders on COVID-19 and prevention strategies. A speaker’s bureau was established to harness and promote these voices to the community. Personal and organizational print and social media platforms were leveraged to produce informative ads and videos. Virtual educational webinars were designed and conducted in partnership with community organizations for further outreach. A targeted messaging campaign leveraging a text messaging opt-in/out platform directed recipients to symptom evaluation tools for COVID-19 and to testing sites and provided additional preventive health reminders. Outputs of this work group are measured by number of community-representative messengers, impact of each media platform, and the attendance of educational sessions.

The data group comprised innovation, health system operations, and research experts working with state and local departments of health to analyze data to establish a strategy to identify hot spots and blind spots of COVID-19 disparities. Partnerships and data agreements to coalesce data streams to produce results are key outputs for the work group to design a dashboard for ongoing monitoring and analysis. This information can be leveraged to design a tailored intervention based on the needs of the identified population of focus, and it was used to develop the text messaging campaign for targeted recipients. Dashboards continue to raise awareness regarding COVID-19 disparities and can be monitored for the closure of the identified disparity gap.

Shared learning of the experiences of mobilizing health care to respond to the community during the pandemic and health disparities research was important and required its own goals to ensure accountability to the academic mission. Research outputs can be measured by the number of publications and grant-supported projects addressing these specific topics.

The final component of the framework required engaging trusted community partners to ensure wider usage of COVID-19 programs, testing, treatment, vaccination, and interventions among economically or socially marginalized populations experiencing disadvantage. We leveraged a parallel effort to establish a community partners advisory board for the work of the collaborative and aid community-based intervention research. A customized email account was created to manage communication to potential community partners. The members of each work group reached out to their personal and professional networks, which represent a diverse group associated with communities experiencing disadvantage (Figure). Colleagues from academic, health care, and community settings were contacted and given a call to action memorandum informing them of the need to support communities most disproportionately affected by COVID-19 and requesting their immediate support. The memorandum outlined the context of the problem and potential solutions. Memo excerpt:

Emory Healthcare has formed a COVID-19 task force to address the health disparities that exist in vulnerable and underserved communities. As part of this task force, the research collaborative is pursuing grant opportunities. We are proposing to rapidly identify hot spots in vulnerable communities in the state of Georgia, to facilitate the connection of these communities to local prevention and treatment resources. We will do this through deployment of our existing community partnerships, our COVID-19 Symptom Check tool, and by delivery of a multi-level intervention targeted at leaders and individual community members. Our approach will be iterative and use community-based participatory implementation research methods. We envision this effort evolving into a national program for rapid identification of COVID-19 hot spots. Currently, we are seeking community partners and advisory board members that are interested in supporting our proposed efforts.

**Figure:**
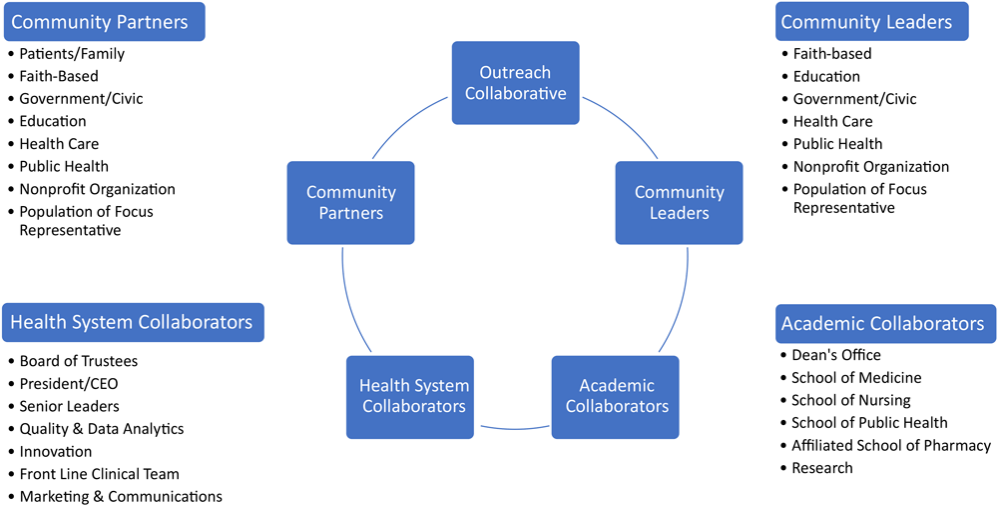
Outreach collaborative key representatives in a framework for mobilizing health care to respond to the community within the COVID-19 pandemic. Abbreviation: CEO, chief executive officer.

### Coalition response

Within 5 days of the call to action memorandum being sent, we received commitments from 36 individuals statewide. Support was received from individuals recovered from COVID-19 (n = 4) and representatives from academic centers (n = 6), health care organizations (n = 6), business and community groups (n = 15), and civic and education agencies (n = 5). These supporters became our community partners. All community partners were asked informally to commit to a 2-year period of involvement. A multiprong virtual communication strategy was used to engage community partners, given social distancing guidelines, including 1) advisory meetings; 2) developing an organizational structure that gives community partners equal decision‐making abilities and virtual attendance at research meetings; and 3) fostering bidirectional learning opportunities through virtual chat platforms. Surveys were then administered to help identify themes of community concerns and barriers, which were used to inform discussion and set common goals.

Individuals who recovered from COVID-19 were asked to provide details of their journey to inform the work and direction of the group. An engagement survey was distributed to partners for feedback on strategies and interventions that require support to mitigate disparities in COVID-19 communities of focus (Box 1). We also surveyed key informants and community partners (Box 2). We were interested in learning their concerns, ideas, and perspective as the team worked toward designing and implementing methods to effectively reach communities disproportionately affected by COVID-19 and other chronic illnesses. Engagement and time commitment preference were also assessed to inform the best way to communicate with the community partners advisory board.

**Box 1. Inteview Questions for Key Informants**

What was your personal experience with COVID-19?

How did this affect your interactions with family and friends?

Where did you have your COVID-19 testing performed? Was there information within your community to help guide you through the process?

Did you have personal experiences during your COVID-19 illness that you felt were different because you are from a minority racial group?

Do you know where/how you contracted COVID-19?

What resources, if any, did you use in your community to obtain more information about COVID-19?

What do you think can be done differently to help underserved and minority populations in Georgia who have been hard hit by COVID-19?

What resources do you think would have been the most helpful for you (or anyone else) diagnosed with COVID-19?

What else would you like to share?

**Box 2. Survey Questions for Community Partners**

Description of the community you represent.

What are the top 3 concerns you have relative to the impact of COVID-19 in the communities you represent?

What do you consider to be the greatest barrier to COVID-19 testing in your community?

What do you consider to be the 3 greatest barriers to masking and other COVID-19 disease prevention efforts in your community?

What messages regarding the COVID-19 pandemic are most important for your community to hear?

Who are the most effective leaders/community partners to deliver trusted messages to the communities you represent?

Would your organization be interested in partnering with Emory to provide free consultations/educational seminars regarding COVID-19? If yes, what kind of consultation would be most helpful?

How would you like to be involved in the Emory Collaborative effort?

Would you be interested in having a meeting (videoconference) with the collaborative and other community partners to further discuss initiatives?

Twenty-six community partners responded to the survey (response rate, 72%). More than half (65%, n = 17) of the partners were interested in partnering with us to provide free consultations or educational seminars regarding COVID-19. Community partners shared a desire to have access to health care consultants for education on COVID-19–related topics. In response, we formed a speaker’s bureau that could be shared with community partners where they could select speaker packages for sponsored events. We also plan to host several education forums that will allow community partners to network. Topics for future forums include risks and benefits of returning children to school in the state of COVID-19 spread, how to engage with others who will not follow the COVID-19 spread prevention recommendations, the impact of physical distancing on social isolation experienced during COVID-19, COVID-19 “Truth and Lies” addressing misconceptions of who is affected by COVID-19, and promotion of our new normal in COVID-19 times — what can we do safely.

Additionally, 58% (n = 15) of the community partners expressed that COVID-19 testing and access to health care were major concerns for their communities, with testing availability being the greatest barrier to testing. A few partners (12%, n = 3) expressed fear of government documentation as a barrier to testing. After the team reviewed the survey results, 2 discussion sessions were held with community partners to further discuss mobilization and next steps. The following themes emerged from these discussions: aging community, connecting the community, access to health care, patient/family experience, staying connected to health care providers during the pandemic, and COVID-19 vaccine trials. These emerging themes will inform the future directions of community outreach efforts.

Interested community, academic, and health care partners will be added on a rolling basis. To maintain the level of engagement, routine meetings (in-person or virtual) are being held to identify problems and strategize solutions to improve outcomes for communities disproportionately affected by chronic disease. The community partner advisory board also has direct access to medical experts through the established speaker’s bureau. We are frequently communicating with the group through quarterly newsletters to share updates and emails for time-sensitive information.

## Strengths and Weaknesses

The key strength of our framework for mobilizing health care through community engagement lies within the unique nature of the multidisciplinary partnerships that have the capacity to reach disproportionally affected communities or those with longstanding issues of mistrust in the health care system (11–14). Leveraging key influencers and community partners is important in addressing many of the health disparities amplified by the COVID-19 pandemic. People with low literacy (and limited health care literacy) need culturally competent, relevant resources and the ability to give their feedback to a trusted community advisor who can help encourage changes to health behavior. Another strength associated with our community partnership model is that it is low in cost to implement, which makes this a feasible approach for low-resource or rural settings. Lastly, once developed, the partnerships can be leveraged to disseminate information about vaccine development and deployment or be a resource in the event of future public health emergencies.

The limitations associated with this framework are similar to those of other volunteer-based programs. Many community partnerships are created based on previously established relationships with individuals and organizations. Community partners could be lost because of a lack of participation in an ongoing collaboration without financial incentives or simply because of a lack of time because of other commitments. To address these weaknesses, partnerships should capitalize on momentum and develop strategies that will ensure community partners remain continually engaged through frequent meetings and directed action items. It would also be fruitful to have community partners serve in leadership roles within the collaborative to promote shared governance and foster trust with the community.

## Implications

This framework for mobilizing health care for community engagement will remain in place beyond the COVID-19 pandemic. The unique nature of these partnerships should be valued and can assist organizations with removing barriers that can hinder community partner and member participation to improve the health status of many groups experiencing disadvantage. Surveys to community members, partners, and groups are critical for assessing their needs to create a purposeful and meaningful agenda to effect change. Connections to the local community will play an increasingly important role as the pandemic continues and can be a way to prevent the spread of misinformation and promote public health best practices. Empowering trusted community leaders is essential to the effective dissemination of a vaccine or information concerning COVID-19 vaccine trials where participation from racially diverse communities remains underrepresented (15). It is important to have appropriate messaging delivered by trusted voices who reflect the community being served.

Having support from the administration of the health care system facilitated the prompt execution of this framework. More specifically, the board of trustees held the collaborative accountable and expected routine reports to ensure that the population of focus received deliverables and resources. We also found that it was meaningful for us to have work groups focusing on messaging and education, data, research, and community partnerships because the output for each group contributed to the goals of another group. We recommend that interested health care organizations adapt this framework to their organizational culture and include these focus areas within their structure to meet their established goals. It is imperative for organizations that have existing collaboratives and efforts addressing health disparities to include key collaborators such as innovators and persons to serve on the front line to engage in the community. In a broader sense, this framework can be used to better prepare economically or socially marginalized communities for future public health emergencies as well as provide insight as the organization looks at the disproportionate share of chronic and other diseases among underrepresented minority and marginalized populations.
